# Supplementary material for: Are carbon emissions trading and green financial instruments synergistic? -Comprehensive quantitative research based on content analysis
Source: PLoS One. 2024 Mar 7;19(3):e0298601. doi: 10.1371/journal.pone.0298601 (PMC10919601; doi:10.1371/journal.pone.0298601)
Supplement: S1 Data — (ZIP) [file pone.0298601.s001.zip › Data/地级市Z_F工作报告.zip!/║■▒▒/╦μ╓▌╩╨/└┤╫╘╣1⁄2╓┌║┼í╛┬φ┐╦╩2╛▌═°í┐_2017.docx]

各位代表：

　　现在，我代表市人民政府向大会报告工作，请予审议，并请各位政协委员和列席人员提出意见。

　　一、2016年和本届政府工作回顾

　　刚刚过去的2016年，我们在省委、省政府和市委的坚强领导下，积极应对经济下行和暴雨洪涝灾害等多重考验，保持定力，凝神聚力，精准发力，扎实推进“圣地车都”和区域性中心城市建设，实现了“十三五”良好开局。

　　（一）经济发展更趋稳健。全市经济稳中有进、进中向好，预计主要指标增速继续好于优于全省、全国平均水平，在全省实现“保五争三”。地区生产总值突破800亿元，达到850亿元，同比增长8.1%。规模工业增加值增长9.3%，高于全省1.4个百分点；全社会固定资产投资1012亿元，增长15%、高于全省1.6个百分点；地方公共财政预算收入45.5亿元，增速高于全省1.5个百分点；社会消费品零售总额441亿元，增长11%。实际利用内联资金270亿元，利用外资1.29亿美元，分别增长11%和12.8%。外贸出口11亿美元，居全省第五位。节能减排等控制性指标全面完成。

　　（二）投资支撑更趋有力。全市新开工项目1570个，增长9.3%，其中亿元以上项目206个，完成投资455.6亿元、增长30.5%。汉十高铁和鄂北水资源配置工程加快建设，齐星万和100兆瓦农光互补电站、先觉庙100兆瓦地面光伏电站并网发电，500千伏区域电网改造项目进展顺利。青岛啤酒20万千升生产线、恒天新能源汽车柔性总装线建成投产，程力5万辆轻卡、金龙集团新型PVC塑胶卷材地板、红星国际广场一期等项目基本完工，中航奥龙应急装备、广同智能装备等项目加快推进。

　　（三）产业结构更趋合理。现代农业加快发展。粮食生产实现丰收，总产达到157.4万吨。新增流转土地15.8万亩，总面积84.6万亩，占家庭承包耕地的45.2%、高于全省平均水平。新增农民合作社398家、家庭农场114家。鼓励资本上山下乡，带动特色农业发展，木本油料种植面积突破60万亩，建成全省最大的油茶、核桃种植示范基地。支柱工业升级增效。实现规模工业总产值1400亿元，增长11.4%；工业用电量增长8.01%，增速高于全省6.09个百分点，居全省第三位；工业产品销售率居全省第一位。专汽产业量质齐升，产值增长15%；成功举办全国应急产业发展大会，启动建设国家专汽质检中心，我市被授予全国专用汽车区域品牌建设示范区。食品工业聚集发展，成功创建省级农业产业化示范园区，天星粮油跻身国家农业产业化重点龙头企业和全国食用油加工企业50强。电子信息产业增速高于规模工业，波导公司实现出口2.5亿美元，成为全市出口规模最大的龙头企业。新兴产业成长兴旺。新能源产业迅猛发展，新增装机40万千瓦，发电量占全省三分之一，首座电动汽车快充站投入使用。电子商务增势强劲，电商交易额110亿元、增长28%，快递业务量增长45%。全域旅游扎实推进，千年银杏谷、西游记公园、大洪山景区入选湖北最具价值旅游品牌，全年接待游客2025万人次，旅游综合收入124.8亿元，分别增长15%和14.2%。创新发展取得成效。主导成立全国专汽技术创新战略联盟，建成湖北省香菇产业技术研究院，新增省级企业研发平台6家，新认定高新技术企业23家、总数达到91家。中国驰名商标、省著名商标分别达到17件和90件。实现高新技术产业增加值95亿元，增长12%，增速居全省前列。

　　（四）改革攻坚更趋深化。全力实施供给侧改革“十大工程”，“三去一降一补”成效明显，企业库存、负债、运营成本不同程度下降，房地产市场保持稳定，中心城区商品房销售面积130.03万平方米、增长65.6%，农民购房占比达到60%。出台降低企业成本七条举措，部分困难企业重组盘活，“退二进三”启动搬迁企业近200家。深化“放管服”改革，“五证合一、一照一码”全面实施，新登记市场主体3.1万户，总数达到15.9万户，注册资本突破1000亿元。建立权责清单，行政权力事项减少23.4%。规范政府债务管理，债务风险总体可控。着力缓解实体经济融资难问题，市金融控股集团组建运营，全市银行业金融机构新增信贷投放103.3亿元，增量存贷比达到57.29%、提高8.36个百分点，余额存贷比48.29%、提高1.15个百分点；全国首批农村土地承包经营权抵押贷款试点工作稳步推进，发放贷款7600万元。直接融资实现重大突破，泰晶科技成为我市首家A股主板上市企业，新增四板挂牌企业8家。争取专项建设基金项目38个、资金25.67亿元，居全省前列。加快发展混合所有制经济，新增混合所有制企业6家，总数达到34家。中心城区集体土地征收补偿标准制定出台，环卫市场化改革全面实施，市直机关事业单位养老保险、公车、城乡医保制度整合等改革进展顺利。

　　（五）城乡发展更趋协调。大力实施重点市政工程建设，麻竹高速浪河互通、白云湖健康公园、传媒大厦、城南污水处理厂一期、随州农副产品物流园一期、城南新区中环路、交通大道综合改造等项目建成使用；氵厥水一桥、涢水一桥、城市公共活动中心综合体、编钟音乐大剧院、高铁站前大道、东护城河治理等项目加快推进。建成474家小餐饮示范店，改造完成东兴、鹿鹤、五眼桥农贸市场，中心城区旱厕全部消灭。村镇面貌加快改善，长岗镇跻身首批中国特色小镇，淅河镇、洪山镇荣登全国千强乡镇榜，桃源村获评全国十佳美丽乡村典型范例，新增10个省级宜居村庄。掀起城乡绿色革命，规划实施中心城区“一轴一环三片”生态工程，完成造林绿化23.6万亩，随南“百里画廊”基本建成，新316国道打造成44公里生态景观走廊。启动编制自然资源资产负债表并开展领导干部专项审计，从严有力整治环保突出问题，全面实行城区“禁鞭”和秸秆“禁烧”，全年空气优良天数多于上年、占比69.4%，PM10均值88微克每立方米、比上年下降14.6%；地表水出境断面水质达标率100%，环境质量企稳向好。

　　（六）社会发展更趋和谐。众志成城抗洪救灾，1870户因灾倒损房屋全部完成重建，水毁设施修复项目完成80%。强力推进精准扶贫，5万人脱贫销号、21个贫困村脱贫出列，易地扶贫搬迁2424户、5130人。创新开展光伏扶贫，新建50千瓦分布式光伏电站223个，争取国家开发银行光伏扶贫贷款18.9亿元。做好就业创业和社会保障工作，城镇新增就业2.3万人，发放创业担保贷款1.3亿元，扶持创业1216人。五项社会保险继续扩面，建筑行业工伤保险参保农民工1.7万人。扎实推进教育事业发展，白云湖学校正式开学，新建、改扩建校舍6.5万平方米、幼儿园31所，高考一本上线率达到20.4%，5名学子被北大、清华录取。县级公立医院改革实现全覆盖，新农合保障水平继续提高，市中心医院顺利通过三甲评审，我市入选首批国家级医养结合试点。全面二孩政策有效实施，出生人口性别比继续下降。提升公共文化服务能力，持续开展送戏下乡等文化惠民活动，新建50个农村综合文体广场，擂鼓墩文物保护区涉军队用地置换方案已与军方达成一致。加快安居工程建设，保障性住房和棚户区改造开工9052套，基本建成7905套，分配入住5623套。住房公积金新增缴存8120人，新增归集7.1亿元，贷款和提取11.3亿元，信息化建设在全省率先达到国家标准。不断创新社会治理，“三民工程”纵深推进，e+为民服务体系覆盖412个村和社区。精神文明建设再上台阶，广水叶星梅家庭被授予第一届全国文明家庭，并受到习近平总书记亲切接见；“光满·爱心红丝带”党员服务队荣获“中国好人”称号，6个先进典型入选“荆楚楷模”，“十星级”创建广泛开展。平安随州建设成效显著，社会治安防控体系逐步完善，化解各类信访积案600多件，安全生产形势保持稳定。

　　（七）政府自身建设更趋高效。依法履行政府职能，自觉接受人大及其常委会的法律监督、政协的民主监督、社会监督和舆论监督，办理人大建议73件、政协提案104件。法治政府建设取得进展，行政事业单位和村、社区聘请法律顾问基本实现全覆盖，广水市被评为全国法治宣传教育先进市。扎实开展“两学一做”，全面推进履职尽责，创新开展行政效率不高、行政执法不公“两不”专项整治，曝光突出问题73个，问责179人，倒逼干部作风持续好转。落实全面从严治党主体责任，层层传导压力，扎实推进党风廉洁建设。积极推进政务公开，“长江云·云上随州”移动政务新媒体平台上线运行。运用大数据开展惠民政策落实情况监督检查，查处了一批侵害群众利益的不正之风和腐败问题。

　　与此同时，新闻出版、文联、社科联、外事侨务、对台、民族宗教、援疆和国防教育动员工作得到新的加强，工青妇、儿童、残疾人、红十字会、无偿献血、老龄和慈善事业有了新的发展，审计、统计、国调、质监、物价、供销、机关事务、公共资源交易管理、档案、保密和地方志工作实现新的进步，邮政、通讯、无线电、石油石化、烟草、民防、消防、水文和气象等工作都取得新的成绩！

　　各位代表，2016年各项工作的顺利收官，标志着市三届人大一次会议提出的主要目标全面实现！“十三五”规划蓝图徐徐展开!五年来，我们应对重大挑战，经受重大考验，取得重大成就，实现重大跨越。

　　——这五年，战略规划引领发展，综合实力持续跃升。我们深化市情认识，明确战略定位，找准发展坐标。“圣地车都”省级战略全面实施，区域性中心城市建设纳入国家大别山革命老区振兴发展规划和湖北“一主两副多极”战略。全市经济倍增发展、跨越赶超，2016年地区生产总值、规模工业增加值、全社会固定资产投资、地方公共财政预算收入分别是2011年的1.6倍、1.7倍、2.75倍和2.74倍。县域经济协调竞进，各县市区主要指标实现翻番，随州高新区进入国家高新区行列，大洪山“佛教名山”建设如火如荼。发展底盘越做越大，累计完成投资3950.3亿元。交通条件大为改善，高速公路万人拥有量全省第二。文化品牌越擦越亮，世界华人炎帝故里寻根节连续八年成功举办，发展软实力显著提升。

　　——这五年，转型升级步履坚实，产业特色持续彰显。我们提升特色优势，多点发力转变发展方式。经济结构更加优化，三次产业之比调整为16：48:36，高新技术产业占比11.4%。实施专用汽车、食品工业千亿元产业推进计划，两大产业占比超过规模工业“半壁江山”。入选省级重点成长型产业集群达到6个，电商、物流等新业态新经济蓬勃发展。建成10个风电场和4个大型光伏电站，新能源装机突破100万千瓦，我市成为名副其实的新能源大市。

　　——这五年，先行先试成果丰硕，开放空间持续拓宽。我们大胆探索，创新实践，全面激活发展动力。商事制度、农村土地承包经营权确权登记颁证、光伏扶贫、医养融合、混合所有制、不动产统一登记等一批“随州经验”在全省、全国推广，我市和曾都区成为全国科技进步考核先进单位。行政审批、医药卫生、食药监管、教育文化、城管执法、国资监管等重点改革纵深推进。多层次资本市场建设收获硕果，湖北银行、交通银行布点随州，企业债券累计融资46.3亿元，挂牌区域股权市场企业达到21家。外向型经济加快壮大，出口总额稳居全省第五，农产品出口稳居全省第一，香菇出口稳居全国地级市首位，出口市场拓展到134个国家和地区。

　　——这五年，城乡建设突飞猛进，生态环境持续优化。我们坚持文化、产业双轮驱动，提升城市能级，协调区域发展，城镇化进程加速推进。中心城区建成区面积扩大一倍，达到80平方公里。常住人口城镇化率49.2%，提高8.9个百分点。“3+3”城市品牌创建圆满收官，“五城同创”扎实推进，随县和广水市城市面貌日新月异，我市被纳入国家新型城镇化综合试点，成功创建国家园林城市、国家森林城市、全国绿化模范城市和全省首个环保模范城市。全市森林覆盖率达到51.8%，远高于全国、全省平均水平，空气质量、水环境质量位于全省前列，生态优势更加显现。随州，每天都有新变化！我们共同的家园，生机勃发、更具神韵！

　　——这五年，各项事业全面进步，民生福祉持续增进。我们坚持兜底民生，共建共享。民生领域累计投入475亿元，占财政支出的79%。城镇、农村居民人均可支配收入年均分别增长10%和11.1%，分别达到24660元和14160元；累计脱贫11.9万人，城镇新增就业突破10万人。覆盖城乡的社会保障、最低生活保障和社会救助体系基本形成，教育文化、医疗卫生、食药安全、社会治理服务等事业协调发展，全市人民的自豪感、获得感和幸福感不断增强。

　　各位代表！五年的成就令人欣慰，五年的经验弥足珍贵。这是省委、省政府和市委坚强领导的结果，是市人大、市政协监督支持的结果，是全市人民团结拼搏的结果!在此，我代表市政府，向全市广大干部群众，向各位人大代表、政协委员，向各民主党派、工商联、人民团体和无党派人士，向驻随部队、武警官兵和政法公安干警，向所有关心、支持和参与随州建设发展的各界朋友，致以崇高敬意和衷心感谢!

　　各位代表！我们也清醒地看到，过去五年的工作，很多是在打基础、补短板。随州发展和政府工作仍然面临一些突出困难和问题。从长期看，我市经济总量不大，产业转型不快，创新链条不完整，民生保障水平不高，发展不够的市情没有根本改变。从当前看，受宏观经济下行影响，不少企业生产经营困难，新旧增长动能接续不足；要素保障压力较大，特别是实体经济融资难问题仍然突出；城市管理不够精细，交通拥堵、物业管理不规范等问题亟待解决。此外，深入解放思想、转变政府职能、根除“四风”顽疾、提高工作质效还需持续用力、久久为功。对此，我们一定直面问题，找准原因，精准施策，积极加以改进。

　　二、今后五年的政府工作

　　未来五年，是随州乘势而上、大有作为的重要战略机遇期。市第四次党代会胜利召开，为未来五年的发展规划了宏伟蓝图，鼓舞人心、催人奋进。面对经济新常态和各地竞相发展的新形势，我们必须抢抓国家和省级战略多重支撑、深化供给侧改革、推进产业转型升级以及我市重大工程、重点项目密集实施等一系列发展机遇，坚持实干兴市，升腾随州气场，探索随州模式，提升随州特色，展现随州作为，努力赢得新一轮发展的主动。

　　今后五年政府工作的指导思想是：高举中国特色社会主义伟大旗帜，深入贯彻落实党的十八大和十八届三中、四中、五中、六中全会精神，深入贯彻落实习近平总书记系列重要讲话精神，深入贯彻落实市第四次党代会精神，按照湖北“建成支点、走在前列”统一部署，坚持以“四个全面”战略布局为统领、以“五大发展理念”为引领，坚持稳中求进的总基调和奋发有为的精神状态，坚持以提高发展质量和效益为中心、以供给侧结构性改革为主线，着力激发创新活力，着力扩大有效投资，着力推进转型升级，着力协调城乡发展，着力深化改革开放，着力保障改善民生，纵深实施“圣地车都”战略，推动经济社会发展迈向更高水平。主要奋斗目标是：经济增长保持“保五争三”态势，地区生产总值在“十二五”末基础上翻一番，人均主要指标达到或超过全国平均水平，城乡居民收入与经济发展同步，社会文明程度显著提高，生态环境质量明显改善，社会治理体系更加完善，区域性中心城市地位巩固提升，全面小康社会顺利实现并迈向新的阶段。突出抓好五个方面工作：

　　（一）持续创新突破，致力厚植特色发展新优势。坚定不移走特色发展之路，努力打造随州品牌。坚持以创新培育特色，大力扶持创新主体，推进科技创新、产业创新、制度创新，建成一批创新平台和科技企业孵化器，力争高新技术产业增加值占GDP比重达到13%以上；全面深化重点改革，强力推进供给侧结构性改革，不断提高供给效益和全要素生产率。坚持以产业提升特色，对接中国制造2025、互联网+，着力构建两大千亿产业引领和多点支撑的现代产业体系，助推新型工业化；优化布局、扩大规模、延伸链条，大力发展现代农业，推进一二三产业融合发展，建成全省重要的农产品加工基地；引导发展“四新”经济，争创国家新能源示范城市。坚持以文化巩固特色，擦亮“炎帝故里、编钟之乡”等文化名片，凸显文化产业支柱地位，增强文化软实力，加快迈向文化强市。突出特色发展导向，完善考核体系，激励各个领域创新、创业、创造，全面推动特色发展。

　　（二）统筹协调并进，致力拓展城乡发展新空间。坚持规划先行、产城融合，推进国家新型城镇化综合试点建设。着力打造智慧城市、海绵城市，大幅提高城市综合承载力，力争常住人口城镇化率达到60%，实现“五城同创”目标，初步建成“双百”城市。推动县域经济做大做强、争先进位，支持随县强核心、美全域、活资源，广水市壮工业、优服务、拓空间，曾都区致力市区一体、建设城北新区、统筹城乡发展，随州高新区集群发展、差异发展、引领发展，大洪山风景名胜区提升核心景区、建好“百里画廊”、发展全域旅游。加快完善城镇基础设施，建设美丽宜居新农村。着力构筑以公路、铁路、航空为骨架的大交通、大动脉，建成鄂北重要的综合交通枢纽城市、物流节点城市。

　　（三）擦亮绿色底色，致力建设生态文明新家园。坚持生态优先、绿色发展，合理布局生产、生活、生态空间，既要金山银山，更要绿水青山。实施绿色产出倍增工程，推进“一轴一环三片”生态建设，在山上再造一个“绿色随州”。加快产业绿色转型，推进节能降耗，发展循环经济，大力倡导绿色低碳生活方式和消费模式。以零容忍的态度坚决向污染宣战，实施大气、水、土壤生态保护行动计划，实现所有集镇自来水厂、污水处理厂、垃圾处理厂规范达标。全力争创国家生态文明先行示范区，让随州更多呈现“高颜值”的碧水蓝天。

　　（四）推进双向开放，致力打造内外联动新格局。抢抓“高铁时代”新机遇，积极参与长江经济带、淮河生态经济带建设，主动对接“一带一路”和湖北自贸区，协同推进大别山革命老区振兴发展。坚持引进来和走出去并重，扩大招商引资，争创国家承接产业转移示范区；多途径开拓出口市场，确保外贸出口突破15亿美元，争创国家级农产品生态原产地保护示范区和汽车及零部件、高新技术产品、茶叶出口基地。

　　（五）促进共建共享，致力满足群众生活新期待。始终坚持民生导向，办好惠民利民之事。把脱贫攻坚作为第一民生工程来抓，确保到2018年建档立卡扶贫对象稳定脱贫，贫困村全部出列，贫困地区造血功能明显增强。大力推进就业创业、教育文化、医疗卫生、健康养老、社会保障等基本公共服务均等化，持续提高城乡居民收入水平和生活质量，力争成为全省最平安的城市之一，努力让发展更有温度、人民的幸福更有质感。

　　三、2017年主要任务

　　千里之行，始于足下。2017年是实施“十三五”规划的重要一年，是供给侧结构性改革的深化之年，也是新一届政府的开局起步之年。开局要有新气象，起步要有新作为。坚持区域性中心城市建设和率先全面建成小康社会目标引领，今年全市经济社会发展主要预期目标是：地区生产总值增长8%，规模以上工业增加值增长9%，固定资产投资增长16%，社会消费品零售总额增长10.5%，地方公共财政预算收入增长8%，外贸出口总额增长8%，实际利用外资增长8%，城乡居民人均可支配收入与经济发展同步，物价形势保持稳定，主要污染物总量减排、环境质量控制等全面完成省考核目标。

　　（一）聚焦聚力全面小康，加快实施扶贫攻坚。层层压实扶贫责任，全力推进脱贫攻坚，确保完成年度目标任务。

　　因地制宜，推进产业扶贫。大力整合项目和资金，集中投入、连片实施，推进开发式、整体性、特色化脱贫。重点实施光伏、旅游、构树、电商等产业扶贫，抓好金融扶贫。扶持发展食用菌、花卉苗木、中药材、果蔬茶、油茶和畜禽水产等特色种养业，推进千年银杏谷全国“景区带村”旅游扶贫示范项目建设。

　　因户施策，定向精准发力。以精准扶贫“十大行动”为抓手，找准“穷根”，开对“药方”，一户一策，动态管理，确保扶贫对象一个不漏。重点在提高贫困群众就业创业能力上下功夫，确保帮扶精准、有效、长久。

　　因势利导，完善公共服务。做好教育、医疗扶贫工作。加快改善贫困地区基础设施，推进交通、水利、电力和互联网建设。将易地扶贫搬迁与完善配套服务、保障长远生计结合起来，确保贫困群众搬得出、稳得住、能致富。

　　（二）聚焦聚力供给侧改革，加快激发动力活力。坚持问题导向，着力增强供给结构对需求变化的适应性、灵活性和动态均衡性，进一步提高改革开放水平。

　　以更大力度落实“五大任务”。继续实施供给侧改革“十大工程”，抓紧抓实“三去一降一补”。探索建立长效机制，落实人地挂钩政策，推广房屋征收货币化补偿，多措并举推动房地产去库存。深化“降本增效”专项行动，严格兑现税费、社保等政策，着力降低企业融资、用工、物流、外贸和制度性交易等运行成本。重视补好基础设施、要素保障、社会事业等方面的短板。

　　以更大决心深化关键性改革。加快政府职能转变，落实权责清单制度，不断提高行政效能。深化“放管服”改革，推进行政审批协同放权，进一步提高审批效率。继续抓好商事制度改革，加快实施“多证合一”，探索一个窗口受理模式，为初始创业者提供便利服务。毫不动摇地支持民营经济发展，鼓励企业与央（省）企重组合作，力争新增混合所有制企业5家，探索建立全省混合所有制改革试点示范区。深化财税体制改革，科学编制中期财政规划，完善国地税征管体制，加强政府债务管理。

　　以更宽视野扩大开放。大力优化出口商品结构，推动技术含量高、附加值高、产业带动性强的产品出口，建好省级食用菌、中药材、生物医药和汽车及零部件出口基地，擦亮国家外贸出口转型升级示范基地品牌，推进随州公用型保税仓建设。支持出口企业巩固传统市场，开发“一带一路”市场，拓展欧美高端市场，支持企业境外投资。

　　（三）聚焦聚力扩大投资，加快筑牢发展底盘。继续把扩大有效投资作为稳增长的主支撑，确保全年新开工亿元以上项目200个以上。

　　加快进度，狠抓项目建设。重点抓好汉十高铁、鄂北水资源配置工程、总装机60万千瓦的风力和光伏发电、区域电网改造等重大项目建设，加快实施黄鹤楼酒业扩能、国药控股、健民叶开泰等重大产业项目。协调支持东风车轮、程力轻卡、青岛啤酒、金龙集团、中航特装等企业投产项目尽快释放产能，积极跟进促成与辽宁忠旺、中车集团、深圳超思维、湖北中储能等企业的合作项目落地。进一步完善项目推进机制，全面推行“五星”管理，下大力气解决融资、用地、征迁等实际困难。

　　把握机遇，狠抓策划储备。紧盯国家投资方向，突出现代产业、基础设施、公共服务三大领域，充实完善市级重大项目库，编制实施三年滚动投资计划。放宽市场准入，充分发挥政府性资金的撬动功能，用好专项建设基金等资金，谋划实施一批PPP项目，千方百计扩大民间投资。

　　重点突破，狠抓产业招商。坚持把招商引资作为“一号工程”，立足做大增量，以先进装备制造、新能源、现代农业、医药化工、文化旅游、商贸物流和电子商务为重点，抓好策划、包装、推介、对接，创新方式，积极开展精准、高效招商，确保引进亿元以上项目40个以上。

　　（四）聚焦聚力转型升级，加快推进新型工业化。坚持改造提升传统产业、培育壮大新兴产业并举，扩总量、提质量、优结构，大力振兴实体经济。

　　以千亿元产业为龙头，提升支撑实力。围绕建成专用汽车、食品工业千亿元产业集群，健全完善政策支持体系。推动专汽产业向新能源、轻量化、智能化等领域进军，建好国家应急产业（专用车）示范基地，建成运营国家专汽质检中心。引导食品工业向品质消费、大健康和精细加工等领域发力。加快壮大电子信息、生物医药、纺织服装、节能环保和战略性新兴产业，突出发展智能装备产业，促进工业化和信息化深度融合。鼓励发展装配式建筑，促进建筑业转型发展。

　　以创新驱动为引擎，提升竞争优势。抓住科技创新“牛鼻子”，实施一批重大科技专项，力争高新技术产业增加值增长13%以上。加强协同创新，深化产学研合作，推动科技成果转化，力争应用成果转化项目30个以上。促进技术、资本、信息、人才等创新要素有效整合与优化配置，支持省专汽研究院、香菇研究院和企业创新平台开展科技攻关。注重扶优扶强，引导企业发扬“工匠精神”，积极开发拳头产品，争当细分行业“单打冠军”。坚持培养、引进、激励并举，吸引更多的优秀人才和创新团队，探索建立人才共享机制。

　　以国家高新区为引领，提升园区效益。科学编制产业发展规划，加快聚集创新产业、培育百亿企业、建设千亿园区，着力打造全市经济第一增长极。大力发展汽车机械、食品工业、风机制造、现代物流、新材料等主导产业，实施一批产业和技改项目。推进府河欢乐世界游乐园、渤海物流城等功能配套项目建设。支持随县、广水、曾都园区管理创新、服务创新和模式创新。

　　（五）聚焦聚力夯实基础，加快构建现代农业体系。坚持重农固本，以农业供给侧改革为动力，夯实现代农业产业、生产和经营三大体系，争创省一二三产业融合发展示范市、国家农业产业化示范园区和全国农产品质量安全市。

　　优化农业结构，促进农民增收。稳定粮食生产，力争粮食总产保持在160万吨左右。加快发展特色农业、效益农业、生态农业，推动粮经饲统筹、农牧渔结合、种养加一体、产供销衔接。继续壮大食用菌、有机茶、蔬菜、优质桃、中药材等特色种植板块，扶持实施马铃薯主食化项目。大力发展木本油料产业，新建基地10万亩，整合加工资源，打造优质品牌。加快转变畜牧业发展方式，突破性发展草食畜牧业。鼓励发展“随州花鲢”等特色水产养殖，推广“稻鱼共生”模式。

　　推进“三权分置”，促进适度规模经营。进一步落实集体所有权、稳定农户承包权、放活土地经营权，规范土地流转管理，发展土地股份合作、托管、代耕等多种经营形式，新增流转面积5万亩，再培育一批家庭农场和农民合作社。积极开展土地承包权有偿退出、经营权抵押贷款等试点。做好第三次全国农业普查工作。

　　创新发展方式，促进农业增效。加快高标准农田建设，实施一批低丘岗地集中连片改造项目。围绕发挥鄂北水资源配置工程的综合效应，加快配套设施建设。推进灌区改造和小型农田水利建设，实现全市水库汛情在线监测管理。引导社会资本上山下乡，培育一批特色农业、生态农业产业园。大力发展智慧农业，加快推进农业物联网试点示范，建设一批互联网+现代农业示范基地。鼓励产地、批发市场探索“基地+社区”“市场+宅配”的鲜活农产品直供模式。

　　（六）聚焦聚力新业态新经济，加快释放后发优势。适应市场倒逼经济转型新趋势，推动各类生产性、生活性服务业加速发展，创造新供给，引导新需求，促进消费升级。

　　突出发展文化旅游业。大力开发特色文化资源，创新发展文化产业，培育文化市场主体，建设文化产业园区。着力提升文化品牌，办好丁酉年寻根节，携手万达影视传媒合拍一部编钟题材的电影。启动建设擂鼓墩考古遗址公园，加快城市公共活动中心综合体、编钟音乐大剧院建设。坚持文化引领、融合发展，积极培育旅游新业态，大力发展乡村旅游，加快发展全域旅游。全力争创5A景区，力争炎帝故里文化旅游区通过验收、大洪山景区通过景观价值评审。大力支持随县省级全域旅游示范区、曾都洛阳生态旅游特色镇建设，支持广水市徐家河旅游度假区建设环库区旅游公路，加快整体开发。加大旅游营销力度，推行旅游 “一卡通”，力争全年接待游客2200万人次、旅游综合收入突破140亿元。

　　突出发展电子商务和商贸流通业。围绕大平台、大数据、大智慧、大仓储、大市场建设，以高新区互联网+创客空间和广水市电商产业园为核心，积极引进电商巨头，集聚电商企业，构建“大电商”格局，加快建设辐射鄂北的电子商务中心。围绕促进内贸流通，推进随州国际物流城、长佳冷链物流园二期等项目建设，积极引进商业综合体。

　　突出发展现代金融业。实施金融领先战略，积极引进各类金融机构入驻，确保随州交通银行上半年正式营业，力争新引进1家股份制银行。新增信贷投放120亿元以上，着力支持实体经济和中小企业。拓宽直接融资渠道，培育、推动优质后备企业上市融资。积极对接长江经济带产业基金，组建市级产业基金并实质性运作，推进市金控集团有效运转和市场化融资，鼓励社会资本发起设立各类股权投资、创业投资等基金。抓好县域金融工程，完善金融服务体系。进一步优化金融生态环境，有效防范金融风险。

　　（七）聚焦聚力“双百”城市，加快建设“神韵随州”。遵循城市发展规律，推进以人为核心的新型城镇化，加快建设生态宜居家园。

　　推进城市功能提升。立足支撑区域性中心城市发展，加快提升功能设施。投资100亿元以上，建设中心城区重点项目90项。实施道路桥梁工程，启动浪河至何店一级公路、南外环一级公路、文帝大道等道路建设，推进氵厥水一桥、涢水一桥、桃园大桥等重点桥梁建设。实施市政设施工程，启动建设高铁站前广场，推进建设4公里地下综合管廊和5平方公里海绵城市。实施便民市场工程，有序搬迁老城区批发市场，改造老旧零售菜市场，配建一批小区便民蔬菜店。实施园林绿化工程，加快涢水城市湿地公园、随城山国家生态公园二期、漂水郊野公园、花溪河公园等建设，确保新增城市绿地80万平方米。

　　推进高效精细管理。健全协调联动机制，加强城管综合执法。坚持重心下移、严格管理，集中治理渣土漏撒行为，大力整治乱停乱靠、占道经营等问题，多措并举缓解交通拥堵。着力打造智慧城管，开通城管APP和“城管通”数字平台，增强城市管理效能。落实《湖北省物业服务和管理条例》，强化职能部门和属地责任，提升小区物业管理服务水平。理顺老旧小区管理体制，着力解决水改、电改和环卫保洁等难点问题。

　　推进美丽乡村建设。加快县乡道改造、危桥加固等基础设施建设，完善村庄垃圾处理、环境管护长效机制，实施村庄道路和庭院绿化美化。加快随南“百里画廊”提档升级，实施乡村旅游线综合治理，着力开发特色民宿、生态观光、健身休闲等项目。大力挖掘乡土文化，建好“荆楚派”特色民居试点。

　　推进环境保护治理。落实领导干部自然资源资产离任审计和环境损害追究制度，加强环境监管网格化管理。提升污水、垃圾处理能力，启动国家重点镇污水处理设施及管网建设，确保高新区淅河污水处理厂和曾都经济开发区污水处理厂建成运行。深入开展环保执法专项行动，巩固扩大中央环保督察成果，重拳打击、公开曝光一批环境违法行为。加强城市周边山体保护，完成“绿满随州”建设任务，切实保障生态环境安全。

　　（八）聚焦聚力民生改善，加快发展社会事业。坚持以发展促民生，集中力量抓好普惠性、基础性、兜底性民生建设。

　　进一步实施就业托底。强化“双创”促进措施，突出做好困难群体、重点人群就业工作，积极开发一批公益性岗位。促进供需对接，拓宽就业渠道，推动劳动力转移就业、就近就业和自主创业，力争新增市场主体2万户以上，其中小微企业1800家以上。

　　进一步完善社会保障。抓好社会保险扩面征缴，推进机关事业单位养老保险改革，全面实施统一的城乡居民医疗保险制度，加强信息平台建设。加大社会救助力度，健全城乡低保、农村五保标准自然增长机制。积极构建社会化养老服务体系，完善配套10家养老机构，新建20家社区居家养老服务中心和100家农村日间互助照料中心。着力改善低收入群众居住条件，持续推进棚户区改造，力争棚改货币化安置率达到80%。

　　进一步发展社会事业。加大投入力度，优化资源布局，协调发展各类教育。重视发展义务教育，加快远程教育信息化建设，着力缓解“大班额”问题；推进学前教育普惠发展，再新建、改扩建一批幼儿园；加快课程课堂教学改革，提升高中教育质量；大力发展现代职业教育，实施产教融合工程，推进市公共实训基地建设。继续深化医药卫生体制改革，加快建立分级诊疗制度，提高医疗质量和服务水平，建成市中心医院新院区，启动市妇幼保健院新院区建设。推进计划生育工作转型，落实全面二孩政策，促进人口均衡发展。大力发展文化事业，加快构建现代公共文化服务体系，广泛开展“三馆一站”免费开放、全民阅读、戏曲进校园等文化活动，加强非物质文化遗产保护传承，举办市第二届体育运动会。巩固省双拥模范城创建成果，不断深化军地共建。

　　进一步创新社会治理。依法推进基层民主，强化城乡网格化管理，不断提高基层治理水平。增强安全发展观念，健全完善排查、预警和处置机制，扎实开展信访积案攻坚，积极化解社会矛盾。牢固树立安全生产红线意识和底线思维，强化责任落实，加强安全隐患治理，严防重特大事故发生。强化食品药品安全监管，做好“双安双创”工作，进一步提升群众满意度。加强应急管理和救灾物资储备，提升防灾减灾能力。推进信用随州建设，加快创建全国文明城市。深化平安随州建设，严厉防范、依法打击盗抢、诈骗、非法融资等违法犯罪行为，确保社会大局持续稳定。

　　四、奋力开创政府自身建设新局面

　　适应新常态、谋求新发展、实现新目标，各级政府责任重大。我们要牢固树立为民、务实、清廉的价值追求，努力建设人民满意的服务型政府。

　　（一）不忘初心，恪守为民之志。始终坚持对党绝对忠诚，切实增强“四个意识”特别是核心意识、看齐意识，坚定不移维护以习近平同志为核心的党中央权威和党中央集中统一领导。深入践行党的群众路线，矢志不渝站稳人民立场，把政策向民生聚焦、服务向民生覆盖、资源向民生倾斜，带着深厚感情和强烈责任为群众办实事、解难题。着力打造“三民工程”升级版，推进由中心城区向随县、广水市社区拓展，不断扩大e+为民服务体系覆盖面，打通联系服务群众“最后一公里”。

　　（二）循法而行，把握施政之要。积极运用战略思维、创新思维、辩证思维和法治思维，着力提升治理能力。深化法治随州建设，实施“七五”普法规划，全面推进科学立法、严格执法、公正司法、全民守法。落实公众参与、专家论证、风险评估、合法性审查、法律顾问和集体讨论决定等行政决策制度。健全监督机制，自觉接受人大法律监督、政协民主监督，强化行政监察和审计监督，主动接受公众监督。健全建议提案办理工作机制，不断提高落实率和代表委员满意率。深化政务公开，积极运用网络问政、电视问政等方式，进一步畅通政府与公众互动渠道，注重做好新媒体环境下舆情引导和回应工作。

　　（三）担当履责，大兴实干之风。更加突出打基础、利长远导向，抓住想干事、敢干事两个关键点，加强履职尽责项目清单管理，重实干、求实效，强化执行落实、争先进位。深入推进作风建设，认真开展“两学一做”，积极践行“三严三实”，深化“两不”专项整治，切实防范和查处各种隐性、变异“四风”问题。坚持正向激励、反向倒逼，健全督查机制，严肃问责违法违纪和不作为、慢作为等行为，推动政务服务提质增效。探索建立“容错”机制，鼓励创新，宽容失误，最大限度地激发、保护干部的干事创业热情。

　　（四）“严”字当头，践行清廉之道。拧紧思想“总开关”，深入推进全面从严治党，把坚定理想信念作为首要任务，正本清源、固根守魂；严格规范党内政治生活，严守政治纪律和政治规矩。抓好领导干部“关键人”，认真落实主体责任和“一岗双责”，公私分明、秉公用权，勤廉自律、以上率下，推进党风廉洁建设，带动政风民风进一步好转。编织制度“防护网”，进一步补齐制度短板，推进惩防体系建设，抓早抓小，严查快处，正风肃纪。管好政府“钱袋子”，严格财政支出、国有资源交易等领域的监管，加强“三公经费”管理，把有限的资金用到惠民生、促发展上。

　　各位代表！民之所望，政之所向。今年，市政府将继续集中力量办好一批民生实事：（1）推进精准扶贫精准脱贫，完成易地扶贫搬迁5998户、12415人，实现47个贫困村脱贫出列、6万贫困人口脱贫销号。（2）实施安居工程，再改造中心城区棚户区5848户；发放住房公积金贷款2亿元，支持农民进城购房落户，促进农民工市民化。（3）改善义务教育办学条件，投入1亿元，新建、改扩建50所义务教育学校校舍4万平方米；确保高新区胜利学校秋季开学、云龙小学基本建成；筹措资金4000万元，资助3万人次家庭困难学生。（4）改善公共交通，调整优化一批公交线路，建设2处公交首末站，完成通村（组）公路350公里，实施农村公路安保工程。（5）完善城乡治安防控设施，加快A类视频探头建设，中心城区新增300个、覆盖100%的行政村。（6）加快随州农副产品物流园二期建设，建成烈山、蒋岗、涢水等3个农贸市场，再建成300家小餐饮示范店。（7）完善休闲文体设施，新建桃园公园和中心城区3处小游园，建成农村、社区文体广场100个和全民健身工程60处。（8）实施安全饮水工程，完成3.86万贫困人口农村饮水安全扶贫任务，确保集镇集中式饮用水水质全面达标。（9）加强水环境改善，推进花溪河渠、东护城河、明珠渠等8条黑臭水体治理，实施城东生态补水和西护城河蓄水工程。（10）大力缓解中心城区交通拥堵，建成汉东东路、明珠东路等城市干道，畅通聚玉街、花溪巷等城市微循环，在学校、医院、商业区附近配建2000个公共停车位。

　　各位代表，激情绘就蓝图，实干成就梦想！让我们紧密团结在以习近平同志为核心的党中央周围，在省委、省政府和市委的坚强领导下，凝心聚力，锐意进取，务实重行，只争朝夕，为建设区域性中心城市、全面建成小康社会而不懈努力，以优异的成绩迎接党的十九大胜利召开！
